# PLGA Nanoparticles for the Intraperitoneal Administration of CBD in the Treatment of Ovarian Cancer: In Vitro and In Ovo Assessment

**DOI:** 10.3390/pharmaceutics12050439

**Published:** 2020-05-09

**Authors:** Ana I. Fraguas-Sánchez, Ana I. Torres-Suárez, Marie Cohen, Florence Delie, Daniel Bastida-Ruiz, Lucile Yart, Cristina Martin-Sabroso, Ana Fernández-Carballido

**Affiliations:** 1 Department of Pharmaceutics and Food Technology, Faculty of Pharmacy, Complutense University of Madrid, Pl Ramón y Cajal s/n., 28040 Madrid, Spain; aifraguas@ucm.es (A.I.F.-S.); galaaaa@ucm.es (A.I.T.-S.); crmartin@ucm.es (C.M.-S.); 2 Institute of Industrial Pharmacy, Faculty of Pharmacy, Complutense University of Madrid, Pl Ramón y Cajal s/n., Universidad Complutense de Madrid, 28040 Madrid, Spain; 3 Department of Gynecology and Obstetrics, Faculty of Medicine, University of Geneva, Rue Michel-Servet 1, 1211 Geneva, Switzerland; Marie.Cohen@hcuge.ch (M.C.); Daniel.BastidaRuiz@unige.ch (D.B.-R.); Lucile.Yart@unige.ch (L.Y.); 4 School of Pharmaceutical Sciences, University of Geneva, Rue Michel-Servet 1, 1211 Geneva, Switzerland; Florence.Delie@unige.ch

**Keywords:** chorioallantoic membrane model, cannabinoids, cannabidiol, gynaecological cancer, nanomedicines

## Abstract

The intraperitoneal administration of chemotherapeutics has emerged as a potential route in ovarian cancer treatment. Nanoparticles as carriers for these agents could be interesting by increasing the retention of chemotherapeutics within the peritoneal cavity. Moreover, nanoparticles could be internalised by cancer cells and let the drug release near the biological target, which could increase the anticancer efficacy. Cannabidiol (CBD), the main nonpsychotropic cannabinoid, appears as a potential anticancer drug. The aim of this work was to develop polymer nanoparticles as CBD carriers capable of being internalised by ovarian cancer cells. The drug-loaded nanoparticles (CBD-NPs) exhibited a spherical shape, a particle size around 240 nm and a negative zeta potential (−16.6 ± 1.2 mV). The encapsulation efficiency was high, with values above 95%. A controlled CBD release for 96 h was achieved. Nanoparticle internalisation in SKOV-3 epithelial ovarian cancer cells mainly occurred between 2 and 4 h of incubation. CBD antiproliferative activity in ovarian cancer cells was preserved after encapsulation. In fact, CBD-NPs showed a lower IC_50_ values than CBD in solution. Both CBD in solution and CBD-NPs induced the expression of PARP, indicating the onset of apoptosis. In SKOV-3-derived tumours formed in the chick embryo model, a slightly higher—although not statistically significant—tumour growth inhibition was observed with CBD-NPs compared to CBD in solution. To sum up, poly-lactic-*co*-glycolic acid (PLGA) nanoparticles could be a good strategy to deliver CBD intraperitoneally for ovarian cancer treatment.

## 1. Introduction

Ovarian cancer is one of deadliest carcinomas in women worldwide [1]. The symptoms are not specific and similar to other non-life-threatening disorders like irritable bowel syndrome, hampering the diagnosis of this pathology. In fact, this neoplasm is usually diagnosed at advanced stages (in 85% of the cases), when the disease has already spread (usually within the intraperitoneal cavity). In fact, this neoplasm shows a low five-year survival rate of around 45% [2,3]. Epithelial ovarian carcinomas represent about 95% of the ovarian malignancies [4]. Therefore, the development of novel therapeutic agents for epithelial ovarian cancer treatment is necessary.

As ovarian cancer cells spread within peritoneal cavity, the intraperitoneal (IP) administration of antineoplastic agents could improve the antitumour efficacy. After IP dispensation, drugs are maintained in close proximity to the target tumour tissues, leading to more rapid and prolonged tumour accumulation [5,6]. However, drug delivery into the peritoneum is limited by their clearance. The use of drug delivery systems could resolve this challenge, increasing the retention of chemotherapeutics within the peritoneal cavity compared to the administration of free drugs [7,8]. Moreover, in the case of chemotherapeutic agents, IP administration is hindered by local toxicity, since high amounts of anticancer drugs are transferred into the peritoneum [9,10]. The use of drug delivery systems that release the drug in a controllable manner is also advantageous and would decrease side effects related to this administration.

Particle size is probably the most important factor that affect the retention and absorption of drug delivery systems within peritoneal cavity [11]. For example, Mirahmadi et al. showed that the higher particle size (from 100 to 1000 nm), the higher retention of the liposomes into the peritoneum [12]. Another aspect that should be considered is the diffusion of the systems within the peritoneal cavity, due to the tendency of ovarian cancer to form peritoneal metastases. While microparticles tend to stay in the vicinity of the administration site, nanoparticles could disseminate more evenly throughout the peritoneum [13]. Furthermore, nanoparticles, due to their small size, could be internalised by cancer cells and let the drug release near the biological target, which could increase the anticancer efficacy [14]. In fact, several nanoformulations have been designed for the IP administration of chemotherapeutics [15,16,17] in the treatment of peritoneal metastases due to their higher efficacy compared to their intravenous administration; thus, a higher amount of the drug is expected to reach the tumour. When nanoparticles are intravenously administered, a part of the drug is released and distributed to other tissues before reaching the tumour. On the contrary, after IP administration of drug-loaded nanoparticles (NPs), the drug would be released within the peritoneal cavity, being relatively easier access to the tumours localised in this area. Finally, it should be noted that IP administration of nanoparticles is, in general, safe, especially when compared to microparticles, which shows a significantly much lower tendency of adhesion formation [18].

In the last decades, cannabidiol (CBD), the main nonpsychoactive constituent of the plant from the genus Cannabis, has emerged as a potential therapeutic tool in oncology as palliative agent, improving the appetite, pain, nausea and vomiting associated with antineoplastics and also as antitumour drug per se, inhibiting the growth and metastasis of tumours [19,20,21]. In general, CBD is a well-tolerated compound (showing a median lethal dose (LD50) of 212mg/kg when administered intravenously to monkeys) [22] with no major safety events, especially compared to conventional anticancer drugs. In fact, several formulations containing CBD are approved for the treatment of epilepsy, spasticity related to multiple sclerosis and cancer pain [23].

Although the anticancer activity of CBD has been stablished in a broad range of tumours, including glioma, breast cancer, prostate cancer, leukaemia and cervical carcinomas, among others, the anticancer activity of cannabinoids, in general, and CBD, in particular, has not been evaluated in ovarian cancer, and our research group is one of the first to study it. Nevertheless, it should be highlighted that cannabinoid receptors (specifically, the type I cannabinoid receptor) are overexpressed in epithelial ovarian cancer, and this overexpression has been associated with the invasive grade of the disease, suggesting the participation of endocannabinoid system in epithelial ovarian carcinomatosis and also the potential use of cannabinoids as anticancer drugs in this neoplasm [24]. Moreover, a recent case report showed a disease response in a patient with low serous ovarian carcinoma, a subtype of epithelial ovarian cancer, who refused standard chemotherapy and was treated with a complimentary alternative therapy that included Laterite and CBD oil [25], reinforcing the potential interest of this cannabinoid in ovarian cancer.

CBD has a low aqueous solubility, which limits its administration, especially by a parenteral route [26]. In fact, the administration of CBD to animals required the use of organic solvents like ethanol and/or dispersing agents (e.g., Cremphor EL or Pluronic F68), increasing the toxicity and limiting the administered dose [27,28]. In this way, nanoparticles are an excellent tool to administer CBD, avoiding the use of organic solvents and dispersing agents [29]. CBD is also an unstable molecule, and its nanoencapsulation could also improve its stability. Moreover, nanoparticles could change drug biodistribution and increase their availability to tumour cells, since lipophilic drugs tend to distribute in fatty tissue. Therefore, the development of polymeric nanoparticles could be a good strategy to dispense CBD IP and to improve its stability and modify its biodistribution. Among all the polymers, poly-lactic-*co*-glycolic acid (PLGA), due to its biodegradability and its FDA and EMA approval for human administration, is one of the most used polymers [30].

The main objective of this work was to design and to develop PLGA nanoparticles loaded with CBD capable of being internalised by ovarian cancer cells to overcome the administration and stability challenges of this drug and to improve its antiproliferative effect. The physicochemical properties of the formulation, physical state of the drug and short-term stability were evaluated. Antitumour efficacy was tested using in vitro and in ovo models of ovarian cancer.

## 2. Materials and Methods

### 2.1. Materials

CBD was obtained from THC-Pharma (Frankfurt, Germany). Poly-(lactide-*co*-glycolic acid-resomer RG^®^ 502 (PLGA-502) (i.v. 0.16–0.24 dL/g) was purchased from Evonik^®^ Industries (Essen, Germany). Polyvinyl alcohol (PVA, Mw = 30,000–70,000), Sigmacote^®^, 3-(4,5-dimethyl-2-thiazolyl)-2,5-diphenyl-2H-tetrazolium bromide (MTT) and Phalloidin Atto-488 were purchased from Sigma-Aldrich (St. Louis, MO, USA). 3,3′-dioctadecyloxacarbocyanine perchlorate (DiO) and 4′,6-diamidino-2-fenilindol (DAPI) were obtained from Invitrogen Molecular probes (Thermo Fisher Scientific, Frederick, MA, USA). Ibidi 8-well plates were obtained from Ibidi labware (Gräfelfing, Germany). HPLC-grade methanol, acetonitrile, dichloromethane (DCM) and dimethyl-sulfoxide (DMSO) were obtained from Fisher Scientific (Frederick, MA, USA). Potassium di-hydrogen phosphate (KH2PO4), disodium hydrogen phosphate dehydrate (Na_2_HPO_4_·2 H_2_O) and Tween^®^ 80 were provided by Panreac (Barcelona, Spain). RPMI Medium 1640 GlutaMAX™-I, Gentamicin (10 mg/mL) Hank’s balanced salt solution (HBSS) and Geltrex^®^ were supplied by Gibco (Life technologies, California, CA, USA). Fetal bovine serum was obtained from Biowest (Nuaillé, France). Cleaved PARP (Asp 214) rabbit monoclonal antibody and anti-rabbit HRP-linked antibody were purchased from cell signalling (Thermo Fisher Scientific, Frederick, MA, USA). GAPDH rabbit antibody (6C5) was obtained from Santa Cruz (Santa Cruz Biotechnology, TX, USA). Demineralised Milli-Q^®^ water (Millipore, Madrid, Spain) was used. All chemicals and reagents were used as received.

### 2.2. Development of PLGA Nanoparticles

Nanoparticles loaded with CBD (CBD-NPs) at 1:5 or 3% (*w/w*) were elaborated by emulsion solvent evaporation technique, using PLGA RG^®^ 502 as polymer. Briefly, PLGA (100 mg) and CBD were dissolved in 1 mL of dichloromethane (DCM) and emulsified in 13 mL of PVA at 1 or 3% (*w/v*) by ultrasonication (Fisher Scientific sonicator, Fisher Scientific, Frederick, MA, USA) in an ice bath for 1–5 min with an amplitude of 90% and a sonication:pause cycle of 40:20 s. Then, particle suspension was stirred for 3 h at 500 rpm at room temperature to allow solvent evaporation. Nanoparticles were collected by centrifugation (15,000× *g* rpm for 35 min) (Beckman Coulter Avanti, Beckman, California, CA, USA) and washed three times with demineralised water in order to eliminate remnants of PVA. Finally, 1 mL of sucrose at 3% (*w/v*) was added as a cryoprotectant, and samples were freeze-dried for 24 h at −50 °C and 0.2 mbar.

For the internalisation experiments, DiO was used as the fluorescent agent, and DiO-labelled nanoparticles (DiO-NPs), at a DiO:PLGA ratio of 0.015:100 mg, were prepared using the aforementioned protocol.

### 2.3. NP Characterisation

#### 2.3.1. Particle Size and Zeta-Potential Measurement

The mean particle size, expressed as volume diameter, polydispersity index (PDI) and zeta potential of nanoformulations (suspended in purified water), were determined by dynamic light scattering using a Microtrac^®^-Zetatrac™ Particle Analyzer (Microtrac Inc., Montgomeryville, PA, USA).

#### 2.3.2. Morphological Examination

The morphology of nanoformulations was evaluated by scanning electron microscopy (SEM), using a SEM Jeol, JSM-6335F microscope (Jeol, Tokyo, Japan). To eliminate the cryoprotectant, nanoparticles were suspended in ultrapure water and centrifuged (15,000× *g* rpm 15 min) three times. Samples were prepared by dropping nanoparticle aqueous suspension (≈100 µg/mL) on a coverslip adhering to a stub and allowing water to evaporate at room temperature for 24 h. Then, dried samples were coated with a 10-nm gold palladium thickness and examined by SEM.

#### 2.3.3. DSC Studies

Thermal analyses of nanoformulations were carried out using a Mettler differential scanning calorimeter (DSC820, Toledo Tech., Zürich, Switzerland) equipped with an aTAC 7/DX instrument controller and aSTARe SW9.10 system software for the data acquisition. The temperature was calibrated using indium standards. Pure CBD, raw PLGA and unloaded and CBD-loaded nanoparticles were analysed. Samples were weighted (≈5 mg) directly into perforated aluminium pans, heated (under a nitrogen flow of 70 mL/min) at a rate of 10 °C/min from 20 to 100 °C, cooled from 100 to 20 °C and heated again up to 100 °C. An empty pan was used as reference. All samples were measured in triplicate. The value of polymer glass transition was calculated in the second heating cycle [31].

#### 2.3.4. Determination of Drug Content and Encapsulation Efficiency

The amount of CBD encapsulated into formulations was determined by HPLC (HPLC, Agilent 1200 series, Agilent Technologies, Santa Clara, CA, USA) equipped with a reverse-phase Mediterranea^®^ C18 (15 × 0.46 cm i.d., pore size 5 μm) (Teknokroma^®^) column. The mobile phase was made of methanol:acetonitrile:water at pH4.5 (52:30:18 *v/v*) at) a flow rate of 1.8 mL/min and an injection volume of 20 µL. The detection wavelength was set at 228 nm.

To determine the content of CBD, lyophilised nanoparticles were dissolved in DCM. CBD was extracted by the addition of mobile phase by vortexing. Then, the samples were filtered and analysed by HPLC. The encapsulation efficiency (EE) was determined by the following equation:

(1) $$EE\;\left(\%\right)=\frac{\mathrm{CBD}:\mathrm{PLGA}\;\mathrm{ratio}\;\mathrm{experimental}\;}{\mathrm{CBD}:\mathrm{PLGA}\;\mathrm{ratio}\;\mathrm{initial}}\times 100$$

#### 2.3.5. In Vitro Drug Release Studies

In vitro drug release studies were performed by suspending nanoparticles (5 mg/mL) in phosphate-buffered saline (PBS) (pH 7.4) containing 0.5% (*w/v*) of Tween^®^ 80 to keep the sink conditions during all the experiment. Samples were incubated in a thermostatic shaking bath at 37 ± 0.5 °C and under a mechanical stirring of 100 rpm for 96 h. At predetermined time points aliquots were removed, centrifuged at 15,000× *g* rpm for 20 min, filtered using a 0.22-µm syringe filter and measured by HPLC.

#### 2.3.6. Stability Studies

The stability of lyophilised CBD-NPs stored at 5 ± 3 °C was evaluated during 3 months. At predetermined time points (2, 4, 6, 8 and 12 weeks), nanoparticles were dispersed in purified water and characterised by the particle size, PDI, zeta potential and CBD loading.

### 2.4. Cell Culture Experiments

#### 2.4.1. Cell Line

SKOV-3 (ATCC^®^ HTB-77™) cells were selected as models of epithelial ovarian cancer due to their high invasiveness and the trend to metastasise within the peritoneal cavity. Cells were purchased from the American Type Culture Collection (ATCC). Cells were grown in RPMI-1640-Glutamax medium supplemented with 10% (*v/v*) of FBS and 1% (*v/v*) of gentamicin and incubated at 37 °C in a humidified atmosphere containing 5% of CO_2_.

#### 2.4.2. In Vitro Cytotoxicity

The cytotoxic activity of CBD in solution (CBD_sol_), unloaded and CBD-loaded nanoparticles was tested in SKOV-3 cells. Cells were seeded into 96-well plates at a density of 1.5 × 10^4^ cells/cm^2^. Twenty-four hours after seeding, the cells were treated for 24 and 48 h with CBD_sol_ (5–50 µM) and the amount of nanoparticles (calculated according drug release studies) that release the equivalent amount of CBD in the same period of time. CBD stock solution was prepared in absolute ethanol and then diluted in complete cell culture medium to reach each concentration. Both unloaded and CBD-loaded nanoparticles were suspended in a complete cell culture medium. Then, the medium of each well was gently discarded; the cells were washed with fresh medium and 100 µL of an MTT solution in complete RPMI medium (0.5 mg/mL) were added to each well. After 3.5 h of incubation, the medium was gently removed, and 100 µL of DMSO were added to each well to dissolve the formazan crystals. The plates were stirred for 10 min, and the absorbance was measured at 570 nm with a microplate reader (Varioskan Flash, Thermo Scientific, Frederick, MA, USA). CBD treatments were added at a concentration in the range of 10–50 µM. Cells treated with complete medium and with Triton-X at 1% served as negative and positive controls of cell death.

IC_50_ was calculated for each treatment for comparison purposes using Compusyn Software (ComboSyn, Inc., Jersey, NJ, USA.) All the experiments were performed in quadruplicate.

#### 2.4.3. In Vitro Cellular Uptake

The uptake of PLGA nanoparticles was determined qualitatively by fluorescence microscopy using DiO-labelled nanoparticles. SKOV-3 cells were seeded into Ibidi 8-well plates at a density of 30,000 cells/well. Cells were treated with nanoparticle suspension (1 mg/mL) in complete RPMI-1640 medium 24 h after seeding and incubated at 37 °C in a humidified atmosphere containing 5% of CO_2_ for 0.5, 1, 2, 4, 6 and 8 h. At each time point, the medium was removed, and cells were rinsed three times with PBS, fixed with 4% paraformaldehyde and stained with DAPI and Phalloidin-Atto 647N. Finally, cells were washed and observed by fluorescence microscopy (Invitrogen™ and EVOS™, Fisher Scientific, Frederick, MA, USA).

#### 2.4.4. Western Blot Analysis

The apoptotic effect of CBD-NPs and CBD_sol_ was investigated by Western blot with the detection of PARP cleavage. Briefly, SKOV-3 cells were seeded at a density of 250,000 cells/well in a 6-well plate. Twenty-four hours after seeding, the medium was removed, and the cells were treated with CBD_sol_ and CBD-NPs at a CBD concentration of 40 µM for 6 and 12 h. Unloaded nanoparticles were also tested. Nontreated and PTX (100 nM)-treated cells were used as negative and positive controls of apoptosis. After the treatments, cells were lysed using RIPA buffer (50 mM Tris-HCl, pH 7.4, 150 mM NaCl, 1% NP-40, 0.1% SDS and 2 mM EDTA). Protein content was measured by Bradford assay, and equal amounts were electrophoresed in SDS polyacrylamide gel and then transferred onto nitrocellulose membranes. Membranes were subsequently immunoblotted with monoclonal-cleaved PARP antibody. A goat HRP-linked antibody was used as the secondary antibody. Specific signal was detected using Amershan ECL Prime Western blotting Detection Reagent (Ge Healthcare, Buckinghamshire, UK). The detection of GAPDH was used as the loading control.

### 2.5. In Ovo Antitumour Activity

The cytotoxicity of CBD in the solution and encapsulated into polymeric nanoparticles was also tested in ovo in SKOV-3-derived tumours formed on the chorioallantoic membrane (CAM) of fertilised chicken eggs [32]. Fertilised chicken eggs were incubated in an automated incubator at 37 °C and 47% humidity in a rotating mode. On egg development day (EDD) 4, a small window was drilled in the eggshells and sealed with tape to prevent desiccation. Then, eggs were placed again into the incubator in a stationary mode. On EDD 8, the window was enlarged, and the CAM were gently scratched to inoculate SKOV-3 cells (2 × 10^6^ cells suspended in Geltrex^®^ matrix per egg). On EDD 11, SKOV-3-derived tumours were formed and surrounded with a silicone o-ring. At this point, the tumour area of each egg was determined using Image J software by surrounding the tumour (tumour area initial). Then, tumours were treated topically with CBD_sol_ (100 µM), unloaded or CBD-loaded nanoparticles (with an equivalent concentration of CBD 100 µM). After the treatments, eggs were placed again into the incubator. On EDD 13.5, the tumour area of each egg was measured as aforementioned (tumour area final). Eggs treated with complete RPMI-1640 medium served as control. All the treatments were added daily from EDD 11 to EDD 13.5. At least 7 eggs per condition were analysed. The tumour growth was determined as follows:

(2) $$Tumour\;growth\;\left(\%\right)=\frac{Tumour\;area\;final\;}{Tumour\;area\;initial}\times 100$$

After the experiments, SKOV-3-derived tumours were collected and paraffin-embedded for histopathological examination. Samples were cut and stained using haematoxylin and eosin.

## 3. Results and Discussion

### 3.1. Design and Development of CBD Nanoparticles

Cannabinoids have attracted a great deal of interest in cancer diseases, and several authors have developed cannabinoid-based nanoparticles to improve its antitumour efficacy, including the development of polymeric nanoparticles [33,34]. However, to the best of our knowledge, this is the first work to develop polymeric nanoparticles loaded with CBD.

Initially, nanoparticles were prepared by a nanoprecipitation technique using acetone as the organic solvent, as with this technique, particles with small size are usually obtained [35]. However, nanoparticles obtained by this method showed a fast CBD release, with more than 80% released in the first 90 min (data not shown). This fast release indicated that CBD was not completely entrapped into the polymeric matrix, which could be attributed to the fast diffusion of acetone from the organic to aqueous phase during nanoparticle elaboration. It is known that nanoparticles need a certain period to be internalised efficiently by the cells, usually 1–2 h in the case of nonmodified PLGA nanoparticles [36,37]. Consequently, with these nanoparticles, most part of the CBD would be lost before the cell uptake. For this reason, the nanoprecipitation technique was rejected, and particles were prepared by the evaporation-extraction method using dichloromethane as the organic solvent.

The characteristics of the formulations obtained by this technique are presented in Table 1. With this method, nanoparticles with a size in the range of 220–260 nm were obtained, which is suitable for their internalisation by cancer cells [36]. Increasing the sonication time (from 1 to 5 min) led to the formation of smaller nanoparticles with a lower encapsulation rate and a faster release of CBD, which could be attributed to a premature degradation of PLGA during the nanoencapsulation process that increases the polymer permeability, leading to the loss of CBD during particle elaboration [38]. Higher initial CBD loading and PVA concentration were also accompanied by a faster CBD release. In the last case, it could be related to the higher amount of PVA that remained associated with the nanoparticle surface [39]. Moreover, the residual PVA impaired the nanoparticle uptake [40], and therefore, the use of lower PVA concentrations would be desirable. However, it should be noted that, in all cases, the percentages of CBD released during the first 90 min (37–72%) were significantly lower than that obtained with the formulations prepared by nanoprecipitation. For these reasons, a sonication time of 2 min, a PVA concentration of 1% and an initial CBD loading of 1.5% were selected as the most suitable parameters to prepare nanoparticles capable of being internalized by ovarian cancer cells with the slowest CBD release.

### 3.2. Characterisation of CBD Nanoparticles

#### 3.2.1. Physical Characterisation

Table 2 gathers the process yield, particle size, polydispersity index and zeta potential of developed nanoparticles. The elaboration protocol reported a process yield above 50% for all formulations. Dynamic light-scattering analyses reported a mono-modal size distribution for all nanoformulations with a mean particle size, expressed as volume diameter, in the range of 228 to 236 nm, being large enough to remain in the peritoneal cavity [41] and small enough to be internalised by ovarian cancer cells. No difference in particle sizes was detected with CBD and DiO incorporation. Regarding the polydispersity index, values higher than 0.1 were obtained in all cases, indicating a polydispersed particle size distribution. Albeit, all the values were lower than 0.2. In general, in the field of polymer-based nanoparticles, values lower than 0.2 are commonly deemed acceptable [42].

Zeta potential was evaluated after the freeze-drying process. As depicted in Table 2, all nanoparticles exhibited a negative zeta potential, related to the uncapped carboxylic groups of the PLGA of the polymeric matrix [43]. Unloaded nanoparticles exhibited a mean zeta potential around −24.7 ± 1.5 mV, similar to the values obtained by other authors for blank PLGA nanoparticles [44,45]. Nevertheless, CBD-NPs exhibited a less negative zeta potential (−16.6 ± 1.2), which is related to drug incorporation. This probably means that, although the drug is entrapped in the polymer matrix, CBD is also exposed partly to the particle surface [46]. Changes in the zeta potential were also detected in DiO-labelled nanoparticles (−28.2 ± 1.5). However, in this case, higher negative values were found.

**Figure 1:**
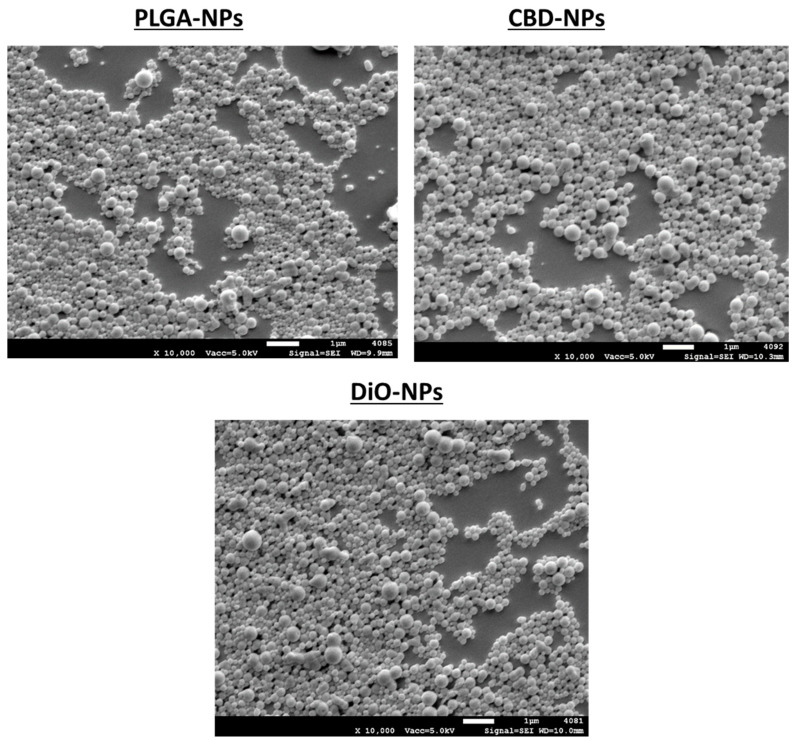
SEM images of unloaded, cannabidiol (CBD)-loaded and 3,3′-dioctadecyloxacarbocyanine perchlorate (DiO)-loaded nanoparticles. PLGA: poly-lactic-*co*-glycolic acid.

Regarding the morphology of nanoparticles, it was evaluated by SEM. As depicted in Figure 1, spherical particles with a smooth and slick surface were obtained in unloaded and CBD-loaded formulations. SEM images revealed that more than one size of population were appreciated, which is in accordance with obtained PDI values. DiO-labelled nanoparticles exhibited an analogous morphology to CBD-NPs.

The DSC thermogram of pure CBD denoted a sharp endothermic peak at 69.68 °C that corresponds to the melting point of CBD and shows an enthalpy value of 75.60 ± 0.95 J/g. This value is pretty similar to that reported in the literature [47]. Nevertheless, in CBD-NPs, this peak was not detected. This indicates that CBD was dissolved into the polymeric matrix.

In respect to the glass transition temperature (Tg), a value of 41.97 ± 1.01 °C was obtained for raw PLGA. A similar value was also detected for unloaded nanoparticles (42.38 ± 0.80 °C). Nevertheless, a slightly lower value was found for CBD-NPs (40.44 ± 0.27), which could indicate a lightly plasticizer effect of CBD. This plasticizer effect of CBD has been previously observed in polymeric microparticles loaded with CBD [48]. However, in the case of CBD-NPs, this effect is so light that the integrity of the formulation is not compromised at the body temperature.

The DSC representation is illustrated in the Supplementary Materials (Figure S1).

#### 3.2.2. Drug Loading, Entrapment Efficiency and In Vitro Drug Release

**Figure 2:**
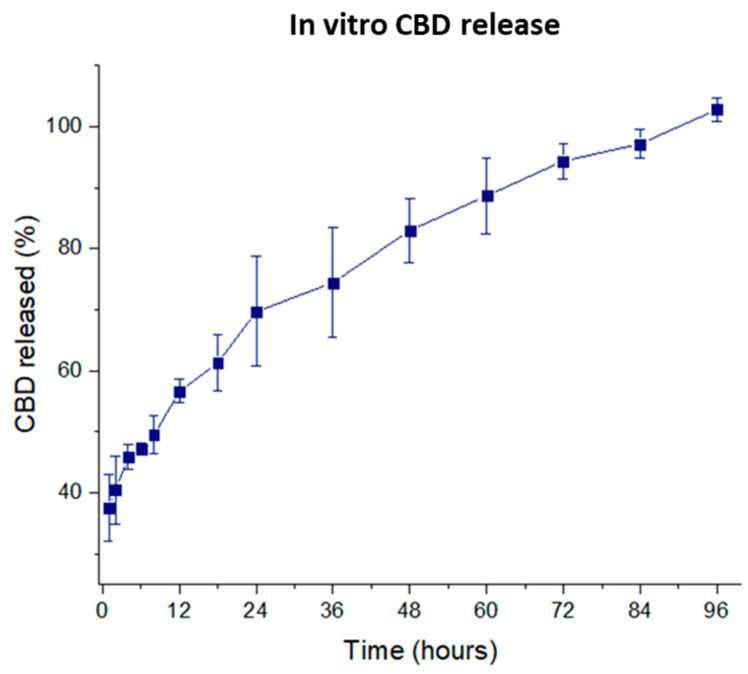
CBD release profile (*n* = 4).

CBD-loaded nanoformulation showed a drug loading of 140.20 ± 6.25 µg CBD/10 mg NPs and a high encapsulation efficiency with values close to 100% (95.23% ± 3.30%). Regarding in vitro drug release, as depicted in Figure 2, a controlled CBD release over a period of 96 h was detected, with 100% of the CBD released by this time. A high burst effect was observed within the first hour, with ≈35% of the CBD released. The CBD release profile was fitted to a zero-order kinetics (r = 0.952) with a CBD release rate of 21.6 µg day^−1^/10 mg nanoparticles. As a consequence, the single administration of this formulation would provide a constant CBD release for 96 h. Due to the acidic extracellular pH value (5-5-6.8) of the tumour cells [49], a faster drug release related to a higher bioerosion of the PLGA could be expected in the tumour microenvironment [50]. For example, Duse et al. found a faster release of curcumin from PLGA-NPs [49] at pH 5.5 compared to 7.4. The effect of the pH on the drug release depends on the contribution of the erosion as the release mechanism. In CBD-NPs, the entire polymer matrix would be eroded due to the low molecular weight of the PLGA-502. An acidic environment would be created in the core of the NPs, at least during the early stages of the degradation, making difficult the buffer effect of the release medium and, in this way, reducing the influence of the pH on CBD release.

As mentioned previously, CBD shows a low aqueous solubility, hampering its administration. The developed formulation showed a suitable strategy to deliver CBD intraperitoneally, making possible the administration of this drug without organic solvents and/or dispersing agents.

### 3.3. Stability Studies In Storage Conditions

**Figure 3:**
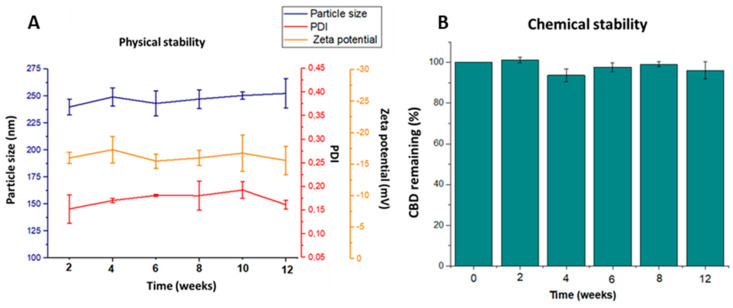
Physical (**A**) and chemical stability (**B**) of CBD-NPs stored at 5 °C during 12 weeks (*n* = 4). PDI: polydispersity index.

In this work, short-term stability studies in storage conditions at 5 °C (selected considering that both CBD and PLGA must be stored refrigerated) over a period of three months were undertaken evaluating the physical and chemical stability of CBD-NPs. As depicted in Figure 3, CBD-NPs remained physically stable for at least three months. Particles were easily dispersed, with no significant changes to either the mean particle size or PDI during the evaluated period. These data suggest that no particle aggregation was produced. This is an important point, since the formation of aggregates would avoid the uptake of nanoparticles by cancer cells. No significant changes in the zeta potential were also appreciated (Figure 3B). Regarding the CBD content, no drug degradation was detected, indicating that CBD encapsulated into the polymeric matrix remained stable for at least three months (Figure 3B).

It should be noted that our research group has found that CBD stability is affected by multiple factors like temperature, oxygen and light. In this way, CBD nanoencapsulation could be advantageous, as CBD-NPs are a stable system that could also protect CBD against oxygen and light.

### 3.4. Nanoparticle Uptake

It is known that the cellular uptake of nanoparticles is influenced by several morphological and physicochemical properties of these systems, including particle size and zeta potential [51]. For this reason, DiO-NPs, used to evaluate the nanoparticle uptake, should exhibit similar properties than CBD-NPs to assume a similar internalisation process. In this work, CBD-NPs and DiO-NPs displayed a similar morphology and an analogous particle size, so a similar internalisation could be expected in these terms. Regarding the zeta potential, it was significantly more negative in DiO-NPs, which could influence the uptake of both formulations. Nevertheless, it has been reported that lower negative values imply a higher uptake. As a consequence, in this way, a similar or even better internalisation could be expected with CBD-NPs (with a lower negative zeta potential) compared to DiO-NPs [52], and therefore, it could be assumed that DiO-NPs are a good strategy to evaluate the cell internalisation of CBD-NPs.

**Figure 4:**
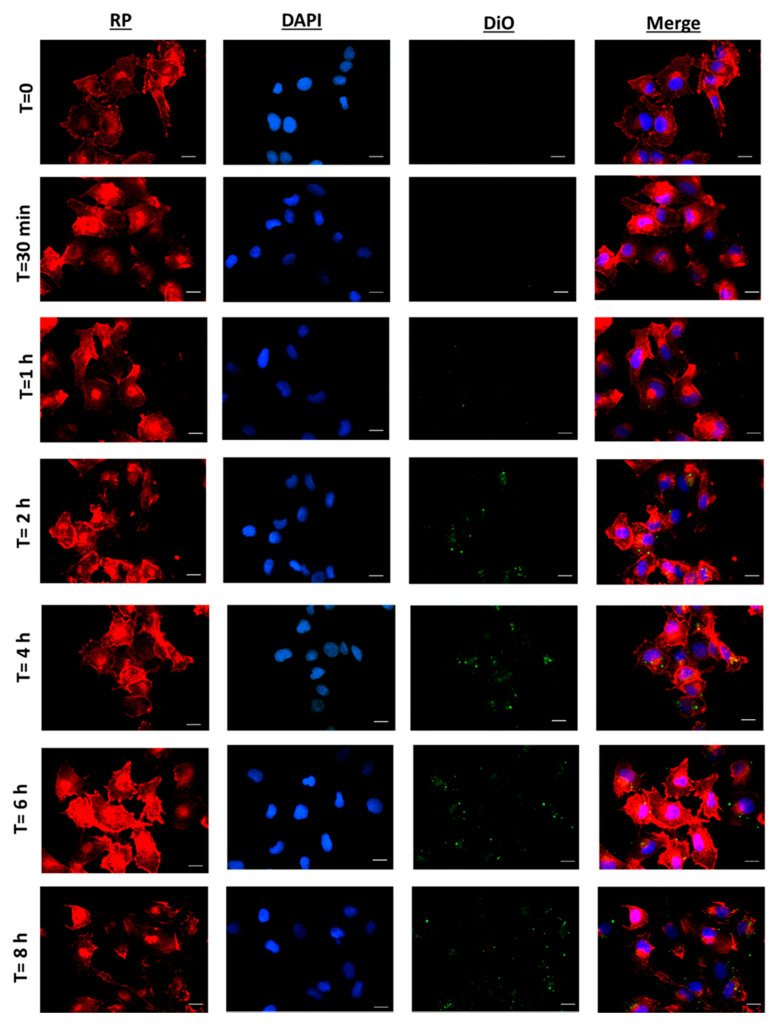
Fluorescence microscopy of SKOV-3 cells incubated with DiO-NPs (at a concentration of 1mg/mL) for 30 min to 8 h. Red images indicate cell cytoskeleton staining (red phalloidin, RP). Blue images indicate nucleus staining (DAPI). Green colour shows DiO-NPs. Uptake experiments were performed in triplicate (*n* = 3). Scale bar: 20 µm.

As depicted in Figure 4, no nanoparticle internalisation was detected during the first 30 min. After one hour of incubation, some uptake was observed. However, NP internalisation started to be significant after 2 h of incubation, increasing up to 4 h. A significant higher uptake was not detected at 6 and 8 h. This indicates that the internalisation of DiO-NPs mainly occurred between 2 and 4 h of incubation. In fact, similar results have also been found by other authors with PLGA nanoparticles loaded with cisplatin and sorafenib, with particles in the range of 230–280 nm and a negative zeta potential, which started to be efficiently internalised by glioma and colon carcinoma cells after 2 h of incubation [37,53].

Due to the similar properties of DiO-NPs and CBD-NPs, it could be expected that cannabinoid-loaded nanoparticles will be internalised by SKOV-3 cells in a time-dependent manner, which could increase the antitumour activity of this agent.

NPs can enter the cells through several mechanisms, the endocytic pathways (micropinocytosis, clathrin-mediated endocytosis and caveolin-mediated endocytosis) being the most common processes [54]. On the one hand, it has been reported that the uptake of nondecorated and negatively charged PLGA-NPs is clathrin and caveolin-mediated endocytosis independent [55]. On the other hand, it is known that clathrin and caveolin-mediated mechanisms are responsible for the internalisation of NPs up to 200 nm [56]. In this context, the developed CBD-NPs that show a negative zeta potential (−16.6 ± 1.2 mV), and a median particle size above 200 nm (236 ± 12 nm) could be internalised by micropinocytosis instead of caveolin and clathrin-mediated endocytosis.

### 3.5. In Vitro Cytotoxicity

The anticancer activity of CBD has been extensively demonstrated in a broad range of tumours, including cervix cancer and other gynaecological neoplasms [57]. Our research group has been the first to evaluate and to report the antiproliferative activity of CBD_sol_ in ovarian carcinoma (unpublished data). In this work, the antiproliferative activity of CBD_sol_ and CBD encapsulated into PLGA nanoparticles have been compared. The cytotoxic activity of CBD_sol_ is illustrated in the Supplementary Materials (Figure S2).

**Figure 5:**
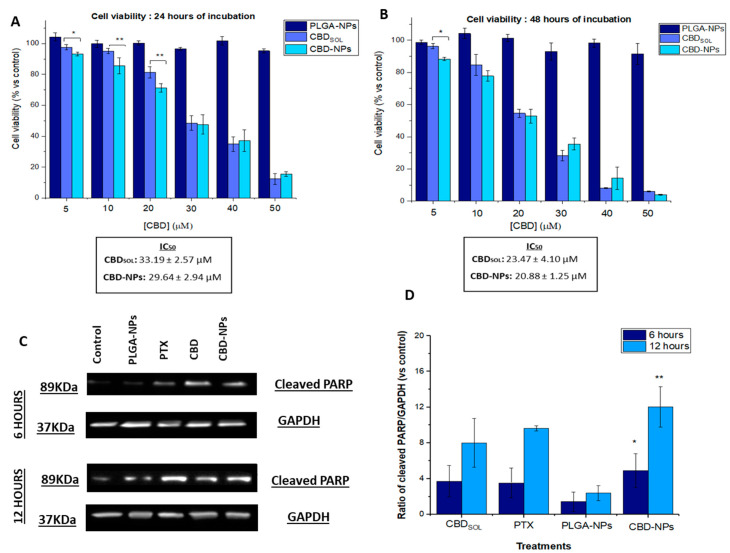
Antiproliferative activity of CBD in solution (CBD_sol_), PLGA-NPs and CBD-NPs in SKOV-3 cells after 24 (**A**) and 48 (**B**) hours of incubation. Western blot analysis of SKOV-3 cells after 6 and 12 (**C**) hours of incubation with CBD_sol_ and CBD-NPs at a CBD concentration of 40 µM, PLGA-NPs and PTX 100 nM. Cells treated with cell culture medium served as control. Ratio of cleaved PARP/GAPDH (**D**). * (*p*-value < 0.05) and ** (*p*-value < 0.01) mean statistically significant differences between PLGA-NPs and CBD-NPs. Antiproliferative studies were performed in quadruplicate (*n* = 4) and Western blot analysis in triplicate (*n* = 3).

As depicted in Figure 5A,B, unloaded NPs were not cytotoxic even at the highest concentration (1.47 mg/mL). Nevertheless, CBD_sol_, as well as CBD encapsulated into PLGA-NPs, inhibited the proliferation of SKOV-3 cells. This inhibition was dose- and time-dependent.

After 24 h of incubation (Figure 5A), statistically significant differences between the cell death induced by CBD_sol_ and CBD-NPs were only detected at low and intermediate concentrations (5–20 μM). In fact, it was observed that, at low CBD concentrations (5–10 µM), CBD-NPs tended to be more efficient than CBD_sol_. This could be attributed to the internalisation of the NPs. Nevertheless, at high CBD concentrations, no significant differences were found between both CBD formulations, probably due to the high cell death rate. After 48 h of treatment (Figure 5B), the same trend was observed at low concentrations; however, significant differences were not found, except for 5 µM. In fact, although differences were not statistically significant (*p*-value > 0.05), lower IC_50_ values were detected in CBD-NPs compared to CBD_sol_ after both 24 (CBD_sol:_ 33.19 ± 2.57 µM; CBD-NPs: 29.64 ± 2.94 µM) and 48 h of treatment (CBD_sol_: 23.47 ± 4.10 µM; CBD-NPs: 20.88 ± 1.25 µM), indicating that the CBD nanoencapsulation trend to improve its antiproliferative effect. Other authors have also found similar results in epithelial cancer cells. Topotecan-PLGA nanoparticles showed the same behaviour in SKOV-3 cells. This nanoformulation also increased the antiproliferative activity of topotecan at lower concentrations, showing a nonsignificant decrease in the IC_50_ value [36]. A higher cytotoxicity was not detected either in A2780 cells (another model of epithelial ovarian cancer) with cisplatin-loaded lipid NPs [58] or pendamidine-loaded PLGA-NPs compared to the free drug. In fact, this latter nanoformulation exhibited even slightly higher IC_50_ values than pentamidine in the solution [59]. This could be attributed to the controlled drug release from these systems. Although CBD-NPs are internalised by SKOV-3, which would increase the antiproliferative effect of this cannabinoid, all administered CBD is not available at the same time.

Apoptosis plays a critical role in determining cell survival. It has been reported that CBD induces programmed cell death in numerous types of tumours, such as breast, prostate or cervix cancer [57,60,61,62]. In this work, the induction of apoptosis has been investigated by Western blotting with the evaluation of PARP cleavage, a key arbitrator in apoptosis. As illustrated in Figure 5C,D, the treatment of CBD_sol_ for 6 and 12 h at a concentration of 40 µM induced PARP cleavage in SKOV-3 cells. Interestingly, similar values were obtained in CBD_sol_ and PTX (used as a positive control of apoptosis)-treated cells (in the Supplementary Materials, Figure S3 depicts SKOV-3 cells treated with both drugs for 12 h). Therefore, this study indicates that apoptosis is responsible, at least in part, for the cell death induced by CBD in these ovarian cancer cells.

Regarding nanoparticle formulations, CBD-NPs-treated cells exhibited PARP cleavage, while unloaded NPs-treated cells did not show a higher expression of cleaved PARP. Thus, NPs did not show a cytotoxic effect in these cells, even after 48 h of incubation. In fact, CBD-NPs exhibited a higher PARP cleavage compared to CBD_sol_. After 6 h of incubation, while CBD_sol_ reported a 3.7-fold induction of cleaved PARP expression, CBD-NPs showed a 4.9-fold induction. This was also evident after 12 h of treatment, with an 8.01 and 12.03-fold induction for CBD_sol_ and CBD-NPs, respectively. However, these differences were not statistically significant. Higher values compared to PTX (6 h = 3.5 and 12 h = 7.6) were also obtained with CBD-NPs (Figure 5D).

The higher PARP cleavage of CBD-NPs compared to CBD_sol_ could be attributed to the internalisation of nanoparticles by the SKOV-3 cells. After 6 h of incubation, DiO-NPs were completely internalised (Figure 3), and it could be assumed that CBD-NPs would show a similar uptake, being efficiently internalised at this time. As a consequence, CBD would be released intracellularly. In fact, other authors have also found that the nanoencapsulation of anticancer agents improves apoptosis induction [63].

### 3.6. In Ovo Antitumour Activity

The CAM model has been proposed as a promising alternative to in vivo assays in cancer research due to the possibility to evaluate the efficacy of novel treatments in a tumour mass without using animals. The chick embryo is not immunocompetent, and cells and tissues from different species could be successfully inoculated [64,65]. In fact, SKOV-3-derived tumours have been implanted successfully in this model [66,67]. Interestingly, these tumours closely resemble the ovarian cancer patient tumours from a histological point of view [68], reinforcing the use of this model to evaluate the activity of novel formulations designed for ovarian cancer treatment in a tumour microenvironment. In fact, this model was recently used to study the anticancer efficacy of biodegradable daunorubicin NPs against epithelial ovarian cancer [69]. In the present work, nanoparticles were designed for IP administration. For this reason, the tumours formed on the CAM were treated topically, since this administration could better mimic the IP route.

**Figure 6:**
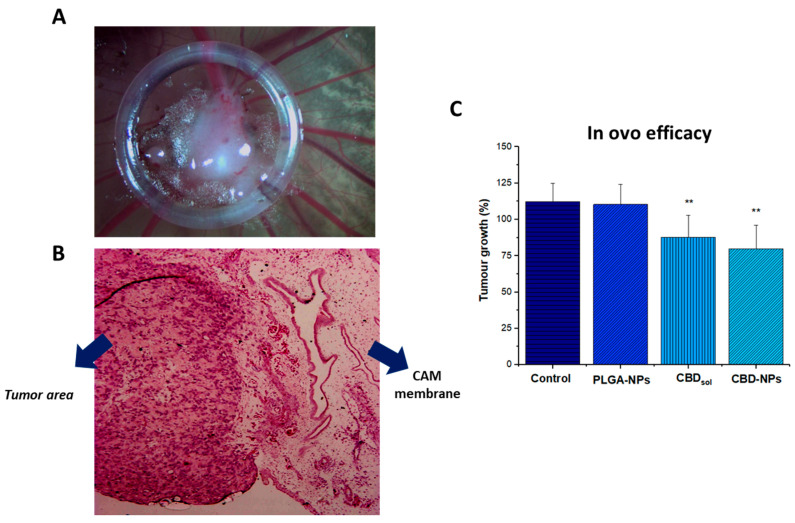
SKOV-3-derived tumour formed on the chorioallantoic membrane (CAM) corresponding with incubation day 11 (**A**), haematoxylin and eosin staining (**B**). Black arrows designated blood vessels. Tumour growth of SKOV-3-derived tumour after several treatments (**C**). ** Statistically significant differences (*p*-value < 0.01) compared to the control (cell culture medium). At least seven eggs per condition were used (*n* = 7).

In this work, SKOV-3-derived tumours were successfully formed, as revealed in Figure 6A. The histopathological examination shows that tumour cells invaded the CAM (Figure 6B) to form the tumour mass.

As depicted in Figure 6C, while unloaded NPs were not toxic, both CBD_sol_ and CBD encapsulated into polymeric nanoparticles significantly reduced the tumour growth (*p*-value < 0.01). Although CBD-NPs exhibited a slightly higher growth inhibition compared to CBD_sol_ (1.5- vs. 1.38- fold reduction, respectively), no significant differences were appreciated.

The slightly higher tumour growth inhibition of CBD-NPs observed in this model indicates that this formulation would facilitate the penetration of CBD into the tumour environment, releasing CBD in the target area and, as a consequence, increasing the anticancer activity. Moreover, after intraperitoneal administration, it could be expected that, with this nanoformulation, the biodistribution of the CBD would be modified, increasing the retention time of this cannabinoid within the peritoneal cavity compared to the administration of the CBD_sol_. Consequently, this formulation would be useful in the treatment of peritoneal metastases of ovarian cancer due to the localisation of the drug at the tumour target.

Although, due to the enhanced permeability and retention effect that characterised the tumours, a high tumour accumulation of the CBD would also be expected after the intravenous administration of CBD-NPs, the amount of CBD that could reach cancer cells would be lower compared to the intraperitoneal administration of this formulation, and, in general, a lower anticancer efficacy could be expected. In fact, the use of intraperitoneal chemotherapy improves the overall survival in patients with metastatic ovarian cancer compared to the administration of intravenous chemotherapy [70], indicating the beneficial use of “intracavitary therapies”. In this context, it should be noted that the developed CBD-NPs show a great potential for other intracavitary administration routes like intravitreal or intracranial, where access of the drug could be hampered and where CBD could be potentially used for the treatment of glaucoma and glioma, respectively.

## 4. Conclusions

CBD was successfully encapsulated into PLGA nanoparticles. These CBD-NPs show several advantages over the use of CBD in solution for the treatment of ovarian cancer. Firstly, this formulation avoids the use of organic solvents and/or solubilising agents for the intraperitoneal administration of CBD.

Secondly, nanoparticles show a suitable particle size staying in the peritoneal cavity and being internalised by cancer cells, releasing the drug from an extended period of four days. Thirdly, the new formulation improves CBD stability as a lyophilised powder. Fourthly, it slightly improves the anticancer activity of CBD in both in vitro and in ovo models, being increased apoptosis when the drug is administered as nanoparticles.

Although further studies are necessary, this work evidenced that PLGA nanoparticles could be a good strategy to administer CBD intraperitoneally in ovarian cancer treatment.

## Figures and Tables

**Table 1:** Characteristics of the formulations elaborated by the evaporation-extraction method during the optimisation process (*n* = 4). CBD: cannabidiol, PVA: polyvinyl alcohol and EE: encapsulation efficiency.

Formulation (F)	CBD (%) (*w/w*)	PVA (%)	Sonication Time (min)	Particle Size (nm)	EE (%)	CBD Released at 90 min (%)
F1	1.5	1	1	258 ± 4	92.37 ± 1.25	37.61 ± 3.22
F2	1.5	3	1	247 ± 3	95.22 ± 3.12	49.80 ± 2.13
F3	1.5	1	2	236 ± 12	93.27 ± 3.10	41.32 ± 2.36
F4	1.5	1	5	220 ± 5	78.13 ± 4.01	59.72 ± 5.57
F5	3	1	2	250 ± 10	80.69 ± 6.22	60.22 ± 1.61
F6	3	3	2	240 ± 8	86.71 ± 2.78	68.27 ± 4.68
F7	3	1	5	229 ± 2	78.08 ± 1.98	72.15 ± 2.45

**Table 2:** Size, polydispersity index (PDI), zeta potential and process yield of nanoparticles. The data are the mean of 4 replicates (*n* = 4). PLGA: poly-lactic-*co*-glycolic acid. DiO: 3,3′-dioctadecyloxacarbocyanine perchlorate. NPs: nanoparticles.

Formulation	Size (nm)	PDI	Zeta Potential (mW)	Process Yield
PLGA-NPs	228 ± 8	0.141 ± 0.019	−24.7 ± 1.5	61.8 ± 4.3
CBD-NPs	236 ± 12	0.165 ± 0.009	−16.6 ± 1.2	51.2 ± 6.2
DiO-NPs	230 ± 7	0.175 ± 0.021	−28.2 ± 1.5	62.5 ± 2.3

## Supplementary Materials

The following are available online at https://www.mdpi.com/1999-4923/12/5/439/s1: Figure S1: DSC thermograms of pure CBD, raw PLGA, CBD+PLGA physical mixture, unloaded and CBD-loaded nanoparticles. Figure S2: Antiproliferative activity of CBD in solution on SKOV-3 cells over a period of 96 h (*n* = 4). Figure S3: Images of SKOV-3 cells treated with cell culture medium (control), PTX (100 nM) or CBDsol (40 µM) for 12 h Magnification: 10x.
