# Supplementary material for: PLGA Nanoparticles for the Intraperitoneal Administration of CBD in the Treatment of Ovarian Cancer: In Vitro and In Ovo Assessment
Source: Pharmaceutics. 2020 May 9;12(5):439. doi: 10.3390/pharmaceutics12050439 (PMC7285054; doi:10.3390/pharmaceutics12050439)
Supplement: Supplementary file 1 [file pharmaceutics-12-00439-s001.pdf]

pharmaceutics

Supplementary Materials: PLGA Nanoparticles for the Intraperitoneal Administration of CBD in the Treatment of Ovarian Cancer: in Vitro and in Ovo Assessment

Ana I. Fraguas-Sánchez, Ana I. Torres-Suárez, Marie Cohen, Florence Delie, Daniel Bastida-Ruiz, Lucile Yart, Cristina Martin-Sabroso and Ana Fernández-Carballido

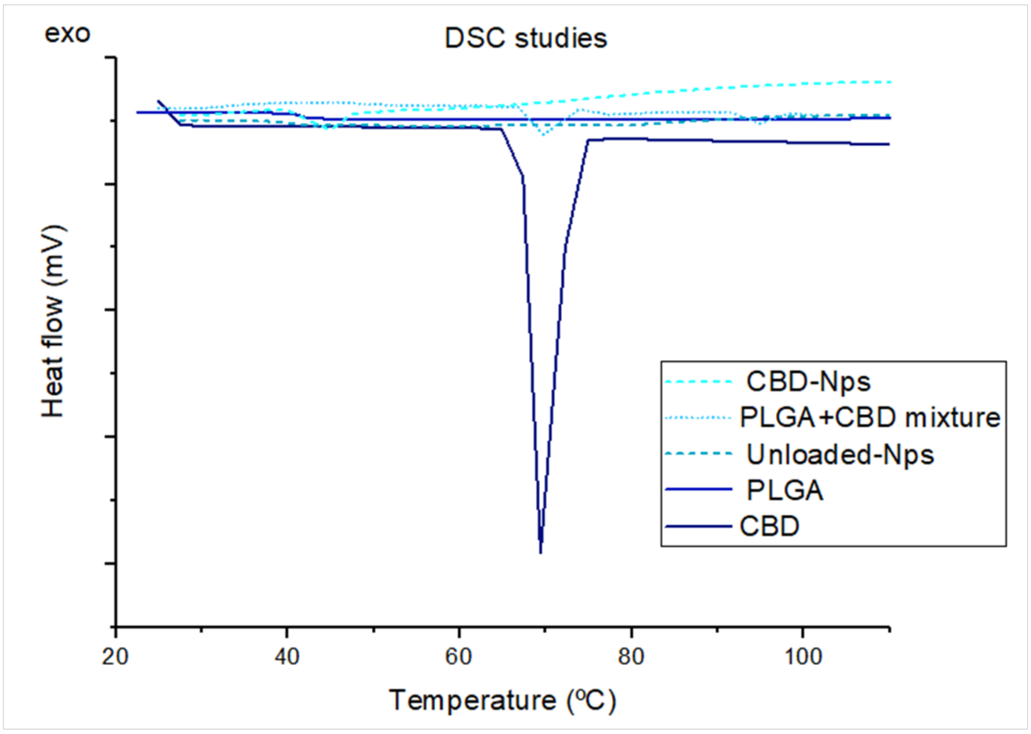

Figure S1: DSC thermograms of pure CBD, raw PLGA, CBD+PLGA physical mixture, unloaded and CBD-loaded nanoparticles.

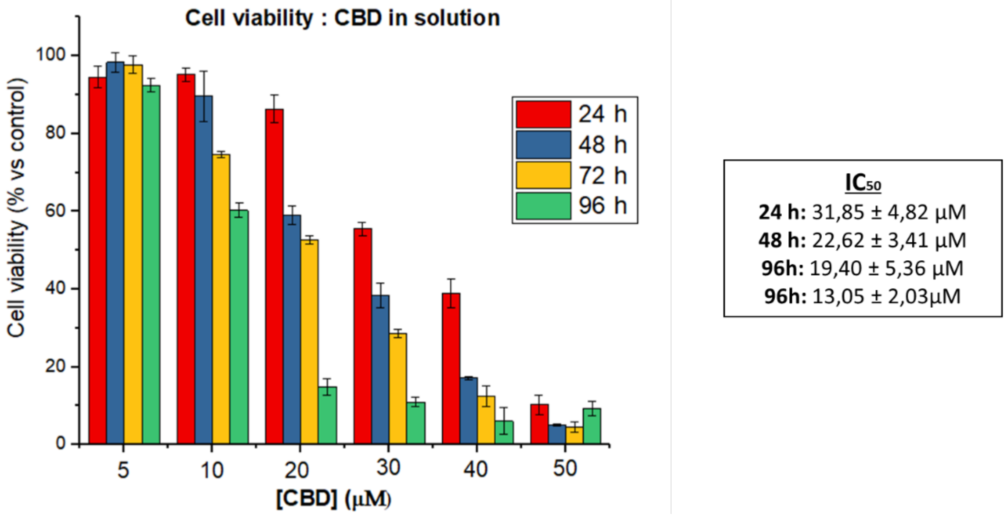

Figure S2: Antiproliferative activity of CBD in solution on SKOV-3 cells over a period of 96 hours (*n* = 4).

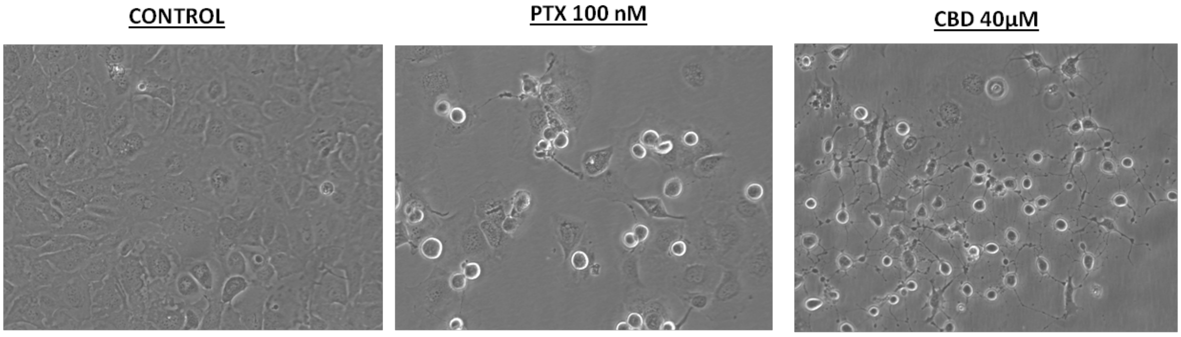

Figure S3: Images of SKOV-3 cells treated with cell culture medium (control), PTX (100 nM) or CBDsol (40 μM) for 12 hours Magnification: 10×.
